# Exploring the Relationship between Noise Sensitivity, Annoyance and Health-Related Quality of Life in a Sample of Adults Exposed to Environmental Noise

**DOI:** 10.3390/ijerph7103580

**Published:** 2010-10-11

**Authors:** Daniel Shepherd, David Welch, Kim N. Dirks, Renata Mathews

**Affiliations:** 1 Auckland University of Technology, Private Bag 92006, Auckland, 1142 New Zealand; E-Mail: renmat01@aut.ac.nz; 2 University of Auckland, Private Bag 92019, Auckland 1142, New Zealand; E-Mails: d.welch@auckland.ac.nz (D.W.); k.dirks@auckland.ac.nz (K.N.D.)

**Keywords:** noise, annoyance, noise sensitivity, health-related quality of life

## Abstract

The relationship between environmental noise and health is poorly understood but of fundamental importance to public health. This study estimated the relationship between noise sensitivity, noise annoyance and health-related quality of life in a sample of adults residing close to the Auckland International Airport, New Zealand. A small sample (*n* = 105) completed surveys measuring noise sensitivity, noise annoyance, and quality of life. Noise sensitivity was associated with health-related quality of life; annoyance and sleep disturbance mediated the effects of noise sensitivity on health.

## 1. Introduction

Health is multifaceted and encompasses not only disease and infirmity but also wellbeing [1]. Numerous factors interact to influence health and wellbeing, including biological (e.g., genetic makeup), lifestyle (e.g., diet), and environmental (e.g., air pollution) factors. Noise, defined at the psychological level of description as an unwanted sound, is increasingly being targeted as an environmental factor negatively impacting health. In some contexts noise can elicit annoyance or disrupt sleep in a manner detrimental to health, though the relationship between noise and health has yet to be satisfactorily elucidated [2–4]. Noise standards emphasize noise level as the primary factor in noise-induced health deficits, however, laboratory [5] and epidemiological (e.g., [6]) findings are increasingly challenging this stimulus-orientated approach, and have instead sought to uncover factors associated with the listener that predict health risk (for reviews see [7,8]).

**Figure 1.**
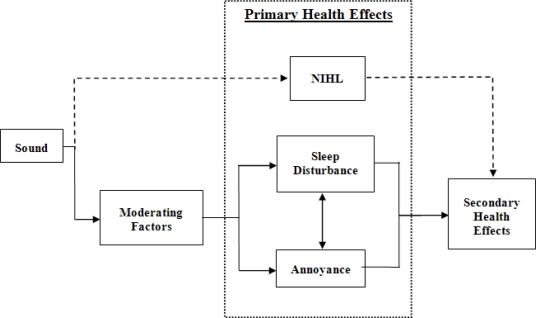
Model detailing how noise might compromise health. The dashed lines indicate the physical effects of noise, which include Noise Induced Hearing Loss (NIHL), while the solid lines represent the non-physical effects of noise. The box labeled “moderating factors” represents the cumulative effect of traits, contextual factors, and noise parameters (e.g., amplitude modulation). Annoyance and sleep disruption act as mediators between predisposing factors and secondary health effects (e.g., health-related quality of life or disease).

Figure 1 is a schematic summarizing the relationship between noise and health. Two pathways are evident, the physical (dashed line) and non-physical (solid line) effects of noise. The physical effects of noise describe those noise-induced health deficits that are associated with sound level and frequency, with Noise-Induced Hearing Loss (NIHL) being an example. Health deficits incurred along this pathway may involve either wanted sound (e.g., attending a rock concert) or unwanted noise (e.g., working with loud equipment). The non-physical effects of noise are those which are mediated by psychological or psychophysiological processes.

There is general agreement in the literature that annoyance and sleep disruptions are the likely mediators of noise-induced health deficits (e.g., [3,6]). However, the relative contribution of noise parameters, personal characteristics, and contextual factors has yet to be determined. In relation to annoyance, the literature indicates that only 10 to 15 percent of the variability in ratings can be explained by noise level, arguing against the use of dose-response relationships as the sole basis for noise standards. The remaining variability is likely to be explained by a collection of interacting traits and contextual factors (*viz* moderating factors in Figure 1) including age [9], noise source and attitude to the noise source [10,11], personality [12,13], mental functioning [4], time of day [14] and noise sensitivity [15,16].

Noise sensitivity, considered a stable personality trait that is relatively invariant across noise level [17], is a strong predictor of noise annoyance [15,18,19], and has been correlated with sleep quality [3,20,21]. Stansfeld [15] described two key characteristics of noise sensitive individuals. First, they are more likely to pay attention to sound and evaluate it negatively (e.g., as threatening or annoying) and second, they have stronger emotional reactions to noise, and consequently, greater difficulty habituating. Noise sensitivity has a large impact on noise annoyance ratings, lowering annoyance thresholds by up to 10 dB [18], and a study of individuals exposed to low frequency noise in the workplace showed noise sensitive individuals were more annoyed by a low frequency noise than a broadband reference noise, while noise-resistant subjects reported that both noises were equally annoying [22]. However, while there is a strong correlation between noise sensitivity and annoyance, the correlation between noise sensitivity and noise level is weak, echoing the marginal relationship found between noise annoyance and noise level [3,7].

In this paper, we report data collected from individuals living in the vicinity of Auckland Airport, New Zealand’s largest and most active airport. The survey area is designated a high aircraft noise area exposed to average outdoor noise levels between 60 and 65 dBA LDN. Consistent with the mode of transport effect [23], aviation noise is rated as more annoying than road traffic or rail noise [24], and we selected this area due to the presence of multiple sources of potentially annoying noise including road, rail, and neighborhood noise. In assessing the heath impacts of noise, a variety of outcome measures have been reported in the literature, including annoyance, sleep disturbance, cardiovascular disease, and wellbeing. One approach to health assessment involves a subjective appraisal of Health-Related Quality of Life (HRQOL), using tools measuring health satisfaction, irrespective of objective health status. The WHO [25] reports that noise-induced annoyance and sleep disturbance can, when chronic, compromise positive wellbeing and quality of life. Dratva *et al.* [26] using the Short Form (SF36) health survey, reported a negative relationship between annoyance and HRQOL in relation to road traffic noise. Published literature reviews indicate that HRQOL would be expected to co-vary more with annoyance than with objective noise measurements [7,8,27]. On this basis, we measured noise annoyance and HRQOL in a confined residential area exposed to constant levels of aviation noise. In accordance with the findings of Dratva *et al.* [26], negative correlations would be expected between HRQOL subscales and noise annoyance. Our main aim, however, is to further evaluate the model presented in Figure 1, specifically the relationship between noise sensitivity and health, and the mediating effects of annoyance and sleep. The interest in noise sensitivity arises due to an increasing number of studies indicating that noise sensitivity is the dominant non-acoustical influence of annoyance and sleep disturbance [3,28,29]. Furthermore, other studies have hinted that annoyance may be a mediating variable between noise sensitivity and mental health (e.g., [4]), though this relationship has yet to be conclusively demonstrated [16,27].

## 2. Methods

### 2.1. Participants

The participants were 105 adults residing in a cluster of relatively homogenous housing approximately 2.5 kilometres east of Auckland Airport’s main runway. According to the New Zealand deprivation scores index [30] this area is ranked 9, where deprivation scores range from 1 (least deprived) to 10 (most deprived) and are calculated using census data corresponding to geographical areas containing a median of 90 people. The region in which Auckland Airport is located has the highest number of decile 9 and 10 (*i.e.*, most deprived) areas in New Zealand [30]. The sample area is designated a high aircraft noise area exposed to average outdoor noise levels between 60 and 65 LDN [31]. The demographic profile of the sample is displayed in Table 1.

### 2.2. Instruments

In addition to items requesting demographic information, the survey contained three self-report assessments, providing measures of HRQOL, noise annoyance, and noise sensitivity. Participants were asked to make their ratings with respect to the previous two weeks. Health-related quality of life was assessed using the World Health Organization Quality of Life (short-form) scale, the WHOQOL-BREF. The WHO ([32], p. 1404) defines quality of life as: “an individual’s perception of their position in life in the context of the culture and value systems in which they live and in relation to their goals, expectations, standards and concerns. It is a broad ranging concept affected in a complex way by the person’s physical health, psychological state, personal beliefs, social relationships and their relationship to salient features of their environment”.

Quality of life, as defined above, is a multifaceted concept, and thus the WHOQOL-BREF produces a descriptive multi-dimensional profile of HRQOL, not a single index. The WHOQOL-BREF consists of 26 items divided into four domains: physical health (7 items), psychological wellbeing (6 items), social relationships (3 items), and environmental factors (8 items). There are two additional items probing overall quality of life and self-rated health. All 26 items in the WHOQOL-BREF are rated on a five point Likert-type scale. A low score on any domain or item equates to negative evaluations of that aspect of life, while a high score indicates a positive evaluation. The BREF is well suited to public health use, and the inclusion of environmental items extends the WHOQOL-BREF beyond traditional HRQOL measures which lack such perspective [33]. The WHOQOL-BREF has excellent reliability and validity [34] and the advantage of adopting a transcultural approach to QOL [34].

Noise sensitivity was estimated using the Noise Sensitivity Questionnaire (NOISEQ) scale [35] which measures global noise sensitivity as well as sensitivity for different domains of everyday life: leisure, work, sleep, communication, and habitation. The 35 NOISEQ items were adapted from the Weinstein Sensitivity Scale and Fragebogen zur Erfassung der Indiviuellen Larmempfindlichkeit (*the Individual Questionnaire of Noise Sensitivity*), and reformulated to increase face validity [35]. Each item asks the respondent to indicate their degree of agreement to statements about their responses to noise using a five point Likert-type scale, which we modified from the original 4-point NOISEQ scales [35]. Global noise sensitivity is computed as the average of the leisure, work, habitation, communication and sleep subscales, with higher means indicating greater sensitivity. The work, sleep and communication subscales have been reported to be sufficiently reliable, while the leisure and habitation subscales not nearly so [35,36].

Susceptibility to noise annoyance was assessed using a 12-item questionnaire developed as a composite of items: 5 items were based on Kroesen *et al.* [37] and focused on annoyance due to aviation noise, and 7 items were based on Thorne [38] and assessed annoyance due to other sources of neighborhood noise. Preliminary assessment using Cronbach’s alpha suggested that it was appropriate to combine these items in that the overall alpha was >0.9 and all item-total correlations were >0.4. All 12 items were standardized and summed to create a General Noise Annoyance scale.

### 2.3. Procedure

Surveys were distributed to 350 randomly selected houses in a confined residential area adjacent to Auckland Airport. In this area, houses were of similar age and were constructed from similar materials. Each selected household received two copies of the survey accompanied by an information sheet and a postage-paid envelope to return the survey. Respondents completed the surveys independently in their own time, and no incentives were offered.

### 2.4. Analysis

All analyses were undertaken using the Statistical Package for Social Sciences (v.17). Prior to constructing summated variables any negatively-worded items were re-coded, and means and standard deviations calculated and inspected for evidence of floor or ceiling effects. Cronbach’s alpha was computed for each scale and item-total correlations calculated to assess unidimensionality. Annoyance items were standardized prior to construction of a summated annoyance variable to remove unintended weightings. Modelling was performed using ordinary least squares linear regressions to scrutinize the relationship between Noise Sensitivity and HRQOL (the criterion variable), and the potential mediating roles played by Noise Annoyance and/or Sleep Quality. In the first step Noise Sensitivity was the sole predictor variable, while in the second step Noise Annoyance and/or Sleep Quality were included simultaneously in the models to test whether they mediated the bivariate relationships. Where regression coefficients between Noise Sensitivity and HRQOL measures were reduced by inclusion of the candidate mediator variables, it was taken as evidence consistent with a mediating role of Noise Annoyance or Sleep Quality on the original relationship.

## 3. Results

**Figure 2.**
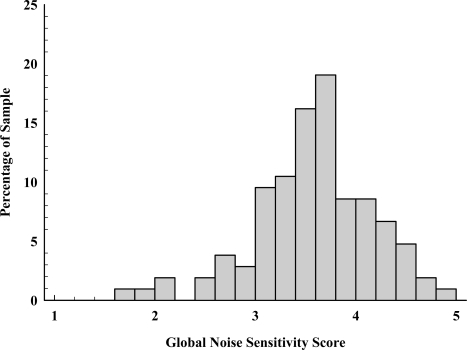
Histogram of Global Noise Sensitivity scores. Global scores are calculated as the mean ratings for all 35 items contained in the NOISEQ. Higher scores represent greater sensitivity to noise.

**Figure 3.**
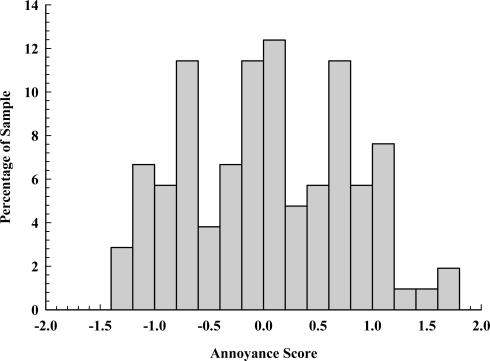
Histogram showing General Noise Annoyance scores. Scores were the mean of 12 standardized noise annoyance items. Of remark is the multimodal nature of the distribution.

All subscales of the NOISEQ, including leisure and habituation, exhibited satisfactory psychometric properties, with means, standard deviations, and Cronbach’s alphas (α_c_) as follows: Leisure (*M* = 3.66, *SD* = 1.49, α_c_ = 0.816), Work (*M* = 3.51, *SD* = 1.3, α_c_ = 0.843), Habituation (*M* = 3.78, *SD* = 1.373, α_c_ = 0.836), Communication (*M* = 3.57, *SD* = 1.36, α_c_ = 0.827), and Sleep (*M* = 3.47, *SD* = 1.62, α_c_ = 0.864). From these subscales, a global noise sensitivity measure (see Figure 2) was computed by computing the average of the five NOISEQ subscales (*M* = 3.58, *SD* = 0.597, min = 1, max = 5, α_c_ = 0.918). The higher the global noise sensitivity score the more noise sensitive the individual, with 51% of our sample having mean scores greater than 3.5. Pearson’s correlation coefficients (*r*) showed that general annoyance (see Figure 3) was positively correlated with all five NOISEQ subscales: Leisure (*r* = 0.343, *p* < 0.001), Work (*r* = 0.354, *p* < 0.001), Habituation (*r* = 0.478, *p* < 0.001), Communication (*r* = 0.273, *p* = 0.005), and Sleep (*r* = 0.412, *p* < 0.001), and also the global noise sensitivity measure (*r* = 0.461, *p* < 0.001).

To afford comparison with other reported aviation annoyance data [6,9,27] the five aviation annoyance items were summed to produce an aviation noise annoyance composite measure having a mean of 13.77 (*SD* = 6.37) and a Cronbach’s alpha of .946. Here a mean close to 5 would indicate no evidence of annoyance towards aviation noise, whilst a mean close to 25 would represent extreme annoyance to such noise. Eighteen individuals scored greater than 20, and thus approximately 17% of participants can be considered severely annoyed. An independent samples *t*-test revealed no gender differences (*t*(103) = −0.771, *p* = 0.443) in overall aviation annoyance score and there were no linear associations with length of residence (*r* = −0.124, *p* = 0.210) or age (*r* = −0.003, *p* = 0.974). On the basis of the nonlinear relationship proposed by van Gerven *et al.* [9], a quadratic model was fitted to the age and aviation annoyance data, with the null hypothesis again supported (*r* = 0.024, *p* = 0.871). To examine the effect of education on aviation annoyance, “university” and “technical” were collapsed to make a higher education variable (*n* = 45), and when tested against those reporting a school-only education (*n* = 59) no differences were found in mean annoyance (*t*(103) = 0.941, *p* = 0.349).

### 3.1. Noise Sensitivity, Noise Annoyance, Sleep Satisfaction, and HRQOL

Table 2 shows that all bivariate associations between measures of Noise Sensitivity and measures of HRQOL were negative (Table 2 (a), Model 1), implying that those with higher sensitivity to noise experienced lower HRQOL. After inclusion of General Noise Annoyance in the models (Table 2 (b), Model 2), the associations between Noise Sensitivity and HRQOL were reduced, implying that Noise Annoyance is a mediator. Note too in Table 2 that the associations between annoyance and the four HRQOL domains, and also self-rated health, reached statistical significance.

According to the literature, sleep quality is often affected by noise, and thus this item was removed from the WHOQOL Physical subscale and included in the modeling as a mediating factor in its own right (Table 2 (c), Model 3). Inclusion of Sleep Quality in the model relating Noise Sensitivity to measures of HRQOL showed that it acted as a mediator as well as introducing independent explanatory power (Table 2 (c)). Simultaneous inclusion of Sleep Quality and General Noise Annoyance in the model (Table 2 (d), Model 4) showed that the relationships between Noise Sensitivity and HRQOL were mediated independently by both General Noise Annoyance and Sleep Quality. The standardized regression coefficient between Noise Sensitivity and the Overall Quality of Life item remained relatively unchanged despite inclusion of Noise Annoyance and Sleep Quality in the model. Furthermore, standardized regression coefficients relating Noise Sensitivity to the Psychological and Environmental aspects of HRQOL remained quite high in Models 2, 3, and 4 despite being attenuated by inclusion of the mediators. Of additional interest is the moderate correlation between the NOISEQ’s sleep subscale and the WHOQOL’s item probing sleep quality (*r* = −0.423, *p* < 0.001).

## 4. Discussion

We undertook exploratory research examining the relationship between noise sensitivity, noise annoyance, and HRQOL. Our results show a broad range of noise annoyance ratings from residents living within a confined area exposed to equivalent levels of aircraft and other sources of neighborhood noise (see Figure 3). Such a finding is inconsistent with the notion that noise level is the main cause of noise annoyance, and instead emphasizes the importance of psychological and contextual factors. The prevalence of severe aviation annoyance (≈17%) found in this study is equivalent to that reported in other Australasian airport studies (see review by Morrell *et al.* [27]), and a model derived from a meta-analysis of European airport studies predict the prevalence of severe annoyance to be between 17% and 25% for aircraft noise between 60 and 65 LDN [24]. According to the WHO Guidelines for Community Noise [39], outdoor noise of 55 LDN is “seriously annoying”. Dose-response curves from 12 European airports suggest that our values are at the lower end of current annoyance estimates, and as such are unlikely to have been overestimated [40]. Note that our aviation annoyance data are consistent with the mode of transport effect [23], with severe annoyance ratings reported in studies on road traffic (13% [26], 9.2% [15]) generally less that aviation and wind turbine noise (25% [41]). Our findings of no significant relationships between aviation annoyance and gender and education are, generally speaking, consistent with the literature (e.g., [6,9,18]), though we found no relationship between aviation annoyance scores and age as reported by others (e.g., [9]). Finally, the lack of association between years of residence and aviation noise annoyance indicates that adverse reactions to noise have not dampened with repeated exposures, that is, there is no evidence of habituation.

There are no reported New Zealand studies measuring noise sensitivity incidence, but our estimate of 50% of individuals being noise sensitive is comparable to international studies (e.g., [15]). Our finding of an association between noise sensitivity and noise annoyance is not novel and adds to a plethora of studies indicating as such (e.g., [3,7]). The correlation we report between noise sensitivity and general noise annoyance (*r* = 0.461) aligns well with those reported elsewhere (e.g., [3,7]). How noise sensitivity influences annoyance has yet be to be described, and the underlying mechanisms of noise sensitivity are not well understood. There are few studies that have investigated the biological basis of noise sensitivity, and genetic studies using monozygotic and dizygotic twins suggest that noise sensitivity has a heritability of 40% [42]. A solitary brain imagining study [43] investigating noise sensitivity showed sensitive individuals had distinctive patterns of brain activity that distinguished them from non-sensitive individuals. Pripfl *et al.* [43] concluded that differences in noise sensitivity most likely reflect a greater strain on cognitive processing. These results concur with previous results suggesting that noise sensitive individuals do not only evaluate a noisy situation as more annoying but also experience higher levels of cognitive strain [44]. Interestingly, on the basis of statistical models, Kroesen *et al.* [37] argue that noise sensitivity does not substantially contribute to annoyance induced by aircraft noise. However, it should be noted that Kroesen *et al.* [37] tested only one of the many proposed models to account for noise annoyance, and furthermore, the analysis may have suffered from spurious relationships amongst empirically-correlated, but theoretically unrelated, variables due to over-specification. In contrast, Fyhri and Klæboe [45], examining the road noise—health relationship and also utilising structural equations modeling, found noise sensitivity to be the dominant variable explaining annoyance.

The standardized regression coefficients we report argue for a negative association between our general annoyance measure and HRQOL domains, and between general annoyance and self-rated health. Literature reviews on the health effects of aircraft noise conducted by Morrell *et al.* [27], and Kaltenbach *et al.* [40], indicate that when the WHO’s definition of health is adopted, the detrimental impact of aircraft noise on health and quality of life are nontrivial. Passchier-Vermeer & Passchier [46] concur, arguing that noise can impair wellbeing and general quality of life, and Dratva *et al.* [26] report an inverse relationship between traffic-related noise annoyance and all SF36 domains excluding general health, especially for individuals who had lived in their homes for six years or less. Thus we reinforce these previous commentaries and the study of Dratva *et al.* [26] and present further quantitative data that noise annoyance can affect HRQOL.

Further to this, we also present evidence that both annoyance and sleep disruption mediate the relationship between noise sensitivity and HRQOL. In relation to sleep it has long been accepted that disrupted sleep reduces psychological wellbeing and effects day-to-day functionality. However, even noise insufficient to cause awakening may cause a brief arousal, with the sleeper moving from a deep level of sleep to a lighter level and back to a deeper level. Because full wakefulness is not reached, the sleeper has no memory of the event but the sleep has been disrupted just as effectively as if wakefulness had occurred. Arousals may be caused by sound events as low as 32 dB(A) and awakenings with events of 42 dB(A) [47]. In one study of aircraft noise, arousals were four times more likely to result than awakenings [48] and were associated with daytime sleepiness [49]. A study undertaken around John F. Kennedy airport in New York, USA, found that 60% of respondents living within 1.6 kilometres of the airport reported sleep disturbance and fatigue [50].

Our use of a cross-sectional design allows us to conclude only that there are associations between noise sensitivity, noise annoyance, and HRQOL, and we cannot confidentially ascribe causal status to any of these three variables. With reference to the health literature it is apparent that current thinking argues that any adverse relationship between noise exposure and physical health is likely to be mediated through psychophysiological processes. Any object or event that an individual perceives as a threat to their safety or to the resting and restorative characteristics of their living environments can be classified as a stressor. Noise is one such psychosocial stressor that can induce maladaptive psychological responses and negatively impact physical health *via* interactions between the autonomic nervous system, the neuroendocrine system, and the immune system [51]. The autonomic nervous system is a mediator of the stress response and expression of stress-related emotion, and consists of parasympathetic and sympathetic branches. Noise sensitivity may be explained by a hypoactive parasympathetic, and a hyperactive sympathetic nervous system. Noise sensitive individuals may delay the termination of sympathetic responses due to an uncoupling of the autonomic nervous system and the amygdala-prefrontal circuits that interpret stressful stimuli and enact the appropriate stress response. The result is that the sympathoexcitatory circuits get caught in a positive feedback loop leading to hyper-vigilance and misattribution that then produce maladaptive cognitions (*i.e.*, annoyance). As the stress accumulates, there is increased activation of the hypothalamic-pituitary-adrenal axis and the sympathetic-adreno-medullary system.

The speculative mechanism discussed above is based on Thayer’s conception of the central autonomic network [52,53], and supports the notion that annoyance can be ascribed causal status in noise-induced health deficits. It must be asked, however, whether poor health itself cannot influence both noise annoyance and noise sensitivity? Our results indicate that while noise sensitivity is partly mediated by annoyance, it is also directly associated with psychological and environmental quality of life. This suggests that psychological wellbeing or environmental factors could potentially mediate noise sensitivity. In relation to psychological wellbeing it has been noted that inhibited restoration in individuals experiencing life stressors or degraded mental health could potentially increase annoyance responses to noise [19]. Causality then is likely to be bi-directional, and potentially create a positive feedback loop in which annoyance and health deficits increase without check. Annoyance can cause degraded health but health itself could potentially amplify annoyance or sensitivity to noise. Thus the model featured in Figure 1 would need to be modified to account for a possible relationship between health and annoyance. Irrespective of causal direction, however, there is still need to consider the effects of sound generators and to position them with care and consideration with respect to the communities hosting them.

### Limitations

First, the sample size was a major limiting factor in the analysis and interpretation of the data. Our small convenience sample likely increased the probability of type I errors by preventing the use of more sophisticated multivariate techniques, and also invited type II errors by providing less than satisfactory power. However, while the findings we report here may be considered somewhat speculative and need to be confirmed with a larger New Zealand sample, they are congruent with findings reported overseas. Future studies capturing more participants would afford the use of structural equations modeling, a more powerful multivariate technique capable of elucidating and testing causal relationships. Second, women were over-represented in the sample (68%), which may have biased the findings in that women may tend to be affected by noise differently from men. Third, we make no attempt to undertake objective measures of noise exposure in this study, noting that while objective noise measurements have had some success in predicting health outcomes using aggregated data, they are severely lacking in predicting individual responses to noise. Dratva *et al.* [26] argue that the ability of subjective annoyance ratings to better account for the individual differences evident in the relationship between noise and health make it a superior marker of the impact of noise on health than noise itself. However, while we make use of outdoor noise contours measured by a professional acoustics company [31], it would have been desirable to undertake indoor noise measurements to further elucidate the relationship between noise and health. Additionally, estimating the time that residents are exposed to the measured noise would likely be an important covariate. Fourth, because we estimated sleep quality using only a single item from the WHOQOL-BREF we can expect greater measurement error around the true values than had we used a composite measure such as the Pittsburgh Sleep Quality Index. Fifth, the use of subjective *versus* objective health measures to detect changes in health due to environmental factors may be viewed as “soft” [27]. Lercher [2] has detailed the methodological challenges of assessing the health impact of noise. Objective outcome metrics such as blood pressure or cardiovascular disease are arguably well defined and easily measured, while noise-induced sleep disruption, stress, and similar subjective symptoms are less easily measured and distinguished from the background levels present in the population. However, objective manifestation of health effects associated with noise-related annoyance may emerge after 5 to 15 years since the onset of exposure [40], whereas subjective appraisals of wellbeing and health suffer no such time lag. Thus for cross-sectional studies as reported here subjective measures are more suitable.

## 5. Conclusions

The subjective experience of annoyance is a common reaction to noise. Different individuals can exhibit different annoyance reactions to the same noise, and these individual differences can be ascribed partly to differences in noise sensitivity. Conceptualized as a stable personality trait, noise sensitivity has no relationship to auditory acuity, instead reflecting a judgmental, evaluative predisposition towards the perception of noise. Our findings suggest that noise sensitivity can degrade HRQOL through annoyance and sleep disruption, though further research is needed to establish causation and afford greater generalizability.

## Figures and Tables

**Table 1.** Demographic characteristics of participants (*n* = 105).

**Variable**	**Category**	**Number**	**Percent**
**Sex**	Male	25	23.8
	Female	72	68.6
	Unspecified	8	7.6

**Age**	18–20	5	4.8
	21–29	9	8.6
	30–39	18	17.1
	40–49	14	13.3
	50–59	28	26.7
	60–69	13	12.4
	70+	17	16.2
	Unspecified	1	1.0

**Ethnicity**	European	51	48.6
	Maori	20	19.0
	Pacific	12	11.4
	Asian	10	9.5
	Unspecified	12	11.4

**Education**	High School	59	56.2
	Technical	25	23.8
	University	20	19.0
	Unspecified	1	1.0

**Occupation**	Employed	49	46.7
	Retired/Sick	22	21.0
	Student	7	6.7
	Unemployed	5	4.8
	On leave	2	1.9
	Housewife	9	8.6
	Other	11	10.5

**Total**		105	100

**Table 2.** Standardized regression coefficients (β) associated with the relationship between Noise Sensitivity and measures of HRQOL (where the Physical subscale has the item reflecting sleep satisfaction removed) modeled using Ordinary Least Squares Linear Regression with (a) Noise Sensitivity alone (Model 1), (b) simultaneous inclusion of Noise Annoyance (Model 2) or (c) Sleep Satisfaction (Model 3), and (d) simultaneous inclusion of both General Noise Annoyance and Sleep Satisfaction (Model 4).

**(a) Model 1 (Simple)**						
	Noise Sensitivity					

Measure	β	*p*-value				

Overall QOL	−0.291	0.003				
Self-rated health	−0.162	0.099				
Physical QOL	−0.238	0.016				
Psychological QOL	−0.349	<0.001				
Social QOL	−0.124	0.231				
Environmental QOL	−0.295	0.003				

**(b) Model 2 (Noise Sensitivity and General Noise Annoyance)**						
	Noise Sensitivity		Noise Annoyance			

Measure	β	*p*-value	β	*p*-value		

Overall QOL	−0.220	0.042	−0.148	0.171		
Self-rated health	0.026	0.807	−0.390	<0.001		
Physical QOL	−0.071	0.500	−0.347	0.001		
Psychological QOL	−0.183	0.073	−0.350	0.001		
Social QOL	0.062	0.581	−0.383	0.001		
Environmental QOL	−0.132	0.210	−0.338	0.002		

**(c) Model 3 (Noise Sensitivity and Sleep Satisfaction)**						
	Noise Sensitivity		Sleep Satisfaction			

	β	*p*-value	β	*p*-value		

Overall QOL	−0.218	0.018	0.353	<0.001		
Self-rated health	−0.076	0.408	0.406	<0.001		
Physical QOL	−0.140	0.115	0.466	<0.001		
Psychological QOL	−0.231	0.004	0.535	<0.001		
Social QOL	−0.029	0.764	0.439	<0.001		
Environmental QOL	−0.182	0.029	0.536	<0.001		

**(d) Model 4 (Noise Sensitivity, General Noise Annoyance, and Sleep Satisfaction)**						
	Noise Sensitivity		Noise Annoyance		Sleep Satisfaction	

	β	*p*-value	β	*p*-value	β	*p*-value

Overall QOL	−0.215	0.037	−0.007	0.946	0.351	0.001
Self-rated health	0.032	0.750	−0.262	0.016	0.321	0.001
Physical QOL	−0.064	0.507	−0.183	0.081	0.406	<0.001
Psychological QOL	−0.171	0.054	−0.150	0.114	0.496	<0.001
Social QOL	0.074	0.478	−0.246	0.029	0.365	<0.001
Environmental QOL	−0.122	0.186	−0.145	0.141	0.490	<0.001

## Abbreviation

HRQOL: Health Related Quality of Life
